# Structural equation modeling of associations between night work and glycemic levels

**DOI:** 10.20945/2359-3997000000147

**Published:** 2019-06-19

**Authors:** Aline Silva-Costa, Lúcia Rotenberg, Valéria Troncoso Baltar, Claudia Medina Coeli, Maria de Jesus Mendes da Fonseca, Enirtes Caetano Prates Melo, Rosane Härter Griep

**Affiliations:** 1 Universidade Federal do Triângulo Mineiro Departamento de Saúde Coletiva Universidade Federal do Triângulo Mineiro Uberaba MG Brasil Departamento de Saúde Coletiva , Universidade Federal do Triângulo Mineiro (UFTM), Uberaba , MG , Brasil; 2 Fundação Oswaldo Cruz Departamento de Epidemiologia e Métodos Quantitativos em Saúde Escola Nacional de Saúde Pública Fiocruz Rio de Janeiro RJ Brasil Departamento de Epidemiologia e Métodos Quantitativos em Saúde , Escola Nacional de Saúde Pública (ENSP- Fiocruz ), Rio de Janeiro , RJ , Brasil; 3 Fundação Oswaldo Cruz Laboratório de Educação em Ambiente e Saúde Fiocruz Rio de Janeiro RJ Brasil Laboratório de Educação em Ambiente e Saúde , Fiocruz , Rio de Janeiro , RJ , Brasil; 4 Universidade Federal Fluminense Departamento de Epidemiologia e Bioestatística Instituto de Saúde Coletiva Universidade Federal Fluminense Niterói RJ Brasil Departamento de Epidemiologia e Bioestatística , Instituto de Saúde Coletiva , Universidade Federal Fluminense (UFF), Niterói , RJ , Brasil; 5 Universidade Federal do Rio de Janeiro Instituto de Estudos em Saúde Coletiva Universidade Federal do Rio de Janeiro Rio de Janeiro RJ Brasil Instituto de Estudos em Saúde Coletiva , Universidade Federal do Rio de Janeiro (UFRJ), Rio de Janeiro , RJ , Brasil

**Keywords:** Night shift work, glycemia, waist circumference

## Abstract

**Objective:**

Different pathways may lead from night work to metabolic diseases, including type 2 diabetes. This study aimed to explore the direct and indirect pathways from night work to glycemic levels, considering the role of physical activity, waist circumference and snacking using data from ELSA-Brasil.

**Materials and methods:**

A structural equation model was used to confirm the pathways from night work to glycemic levels. The latent variable, “glycemic levels”, included fasting glucose, glycated hemoglobin and 2-hour plasma glucose.

**Results:**

A total of 10.396 participants were included in the analyses. The final model showed that among women, night work was associated with increased glycemic levels. A statistical significant association between night work and glycemic levels mediated by waist circumference was observed among women and men.

**Conclusions:**

The association between night shift and glycemic levels can be interpreted as an important step toward understanding the pathways that could explain night work as a risk factor for diabetes using epidemiological data.

## INTRODUCTION

Several epidemiological studies have suggested exposure to night work as a risk factor for type 2 diabetes ( 1 - 4 ). Authors generally point out that the association between work schedule and glucose metabolism may result from the combination of unhealthy diet patterns, physical inactivity, and circadian disruption ( 5 - 7 ).

In line with this discussion, Pan and cols. ( 8 ) showed that BMI could be considered a mediator in the relation between shift work and risk of type 2 diabetes (DM2). Similarly, BMI and waist circumference may function as mediators ( 9 ), since unhealthy behaviors and circadian disruption among night workers may favor increased risk of obesity, insulin resistance, and then DM2 ( 5 , 10 ). A meta-analysis found higher risk of diabetes among shift workers when BMI and physical activity were excluded from regression analyses ( 11 ), suggesting that indirect pathways linking shift work with diabetes may involve these two variables. Therefore, the discussion related to the factors that can act as mediators or confounders variables is relevant. The possibility of testing pathways from night work to glycemic levels with structural equation instead of traditional approach using adjusted regression models is an important step for this discussion. Besides, the heterogeneity in shift work definition and the current or past time of exposure to night work can contribute for the understanding of some inconsistent results in the literature. Additionally, a meta-analysis suggested a stronger association between shift work and DM2 in men than in women ( 11 ), but studies of gender differences are still inconclusive.

In view of the gaps in the literature related to shift work and metabolism, and considering that night shift work is an inherent growing part of 24/7 society, the understanding of the relation between night work and diabetes can benefit from analysis of alterations in glycemic levels. Wang and cols. ( 12 ) suggested that studies should explore these possible mechanisms for the relationship between shift work and diabetes in order to highlight the importance of lifestyle factors as potential mediators. In other words, the understanding of previous or intermediate risk factors for the development of diabetes from shift work exposure is relevant, mainly, to promote clinical interventions.

Accordingly, using baseline data from the Brazilian Longitudinal Study of Adult Health (ELSA-Brasil), this study explored direct and indirect pathways for effects of night work on glycemic levels, considering the role of some health behaviors, physical activity, body weight and snacking.

## MATERIALS AND METHODS

### Study population

The Brazilian Longitudinal Study of Adult Health (ELSA-Brasil) is a prospective cohort study designed to identify risk factors for diabetes and cardiovascular diseases. At baseline (2008-2010), the cohort comprised 15105 civil servants aged 35-74 years from five universities and one research institute in six Brazilian cities. The study was approved by the Research Ethics Committee of each institution involved. All study participants provided written declarations of informed consent ( 13 ).

### Variables

Baseline assessments (2008-2010) included clinical and laboratory measurements and a comprehensive set of questionnaires on sociodemographic, occupational and health characteristics, and followed a rigorous process to guarantee the quality of the data ( 14 - 16 ).

To estimate glycemic levels, a 12-hour fasting blood sample was drawn by venipuncture soon after each subject’s arrival at the clinic to measure fasting glucose and glycated hemoglobin. A 2-hour plasma glucose level, obtained during a 75-g oral glucose tolerance test, was also measured as described in detail elsewhere ( 17 - 19 ).

Weight (kg), height (m), and waist measurement (cm) were collected using standard equipment and techniques. Body mass index (BMI) was defined as weight/height ^2^ ( 18 ). Assessment of snacking, from the semi-quantitative food frequency questionnaire, included consumption frequencies for pizza, hamburger, hot dog, ham/salami/mortadella, fried savories, and soda. The frequency options were: more than 3 times a day; 2-3 times a day; once a day; 5-6 times a week; 2-4 times a week; once a week; 1-3 times a month, and never/almost never ( 20 ).

Work schedule was classified into two categories: day workers (participants with no night-work exposure) and night workers (those whose work arrangement included at least one night shift per week).

Information on age, sex, education, smoking (never, former and current smoker), alcohol consumption (g alcohol/week) and leisure physical activity (none, moderate, high; obtained using the International Physical Activity Questionnaire, IPAQ), and use of medication for diabetes, were also obtained by questionnaire.

For the current analysis, to avoid the influence of prior exposure to night work on health ( 21 , 22 ), all analyses of day workers excluded those with prior night-work experience, as detailed information was not available on the length of time since night work ceased. Similarly, retired workers were also excluded from the analyses ( 23 ). The final study population comprised 10396 current workers.

### Statistical analysis

For descriptive analyses, categorical variables were expressed as percentages, and continuous variables, as mean and standard deviation (SD). Structural equation modelling (SEM) was used to test for pathways from night work to glycemic levels. Unlike traditional regression analyses, SEM is a method using multiple linear equations to include direct and indirect effects, as well as latent variables (i.e., variables not directly observed).

**Figure 1:**
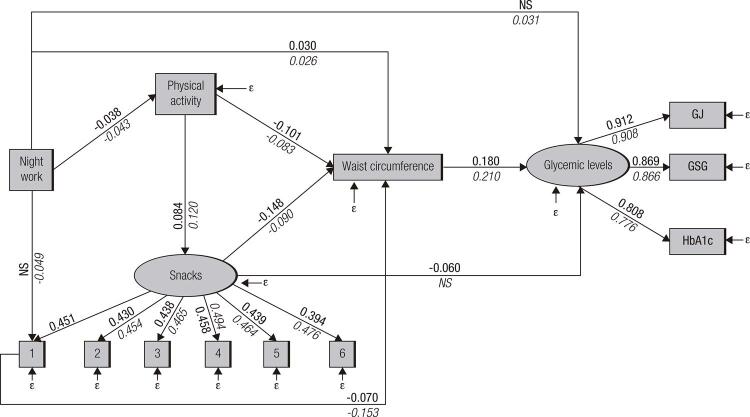
A structural equation model for direct and indirect effects of night work on glycemic levels, by gender: ELSA-Brasil, 2008-2010.

Based on the literature, we tested models including food consumption estimated by diet quality index, waist-to-hip ratio, BMI, job strain, total work hours per week, smoking and alcohol consumption as factors in pathways between night work and glycemic levels. The final model estimated two latent variables: i) Glycemic levels (GLIC), which included glucose drawn from a 12-hour fasting blood sample (FG), glycated hemoglobin (HbA1C) and 2-hour plasma glucose (GSG); and ii) snacking, which included consumption frequencies for pizza, hamburger, hot dog, ham/salami/mortadella, fried savories, and soda (a higher score indicating a healthier diet). Physical activity, waist circumference, and work schedule were also retained in the final structural model ( Figure 1 ).

Values in bold represent standardized coefficients for men; values in italic represent standardized coefficients for women.

WC: waist circumference; GLIC: glycemic levels; FG: fasting glucose; HbA1C: glycated hemoglobin; GSG: 2-hour plasma glucose; 1: soda; 2: pizza; 3: ham/mortadella/salami; 4: hot dog; 5: hamburger; 6: fried savories. NS: non-significant.

Night work was hypothesized to have an effect on glycemic levels (GLIC) indirectly through the mediation of body weight, snacking and physical activity. All regression analyses were adjusted for the potential confounders age, education, and diabetes medication use. Note that the term “effect” used here refers to the association between the variables in the model, as the cross-sectional nature of this study does not allow the directionality of the relations to be determined.

A robust maximum likelihood method (MLR) was used to estimate parameters. Model fit was assessed by comparative fit index (CFI ≥ 0.90), the Tucker-Lewis index (TLI ≥ 0.90), residual mean square error of approximation (RMSEA ≤ 0.06) and its 90% confidence interval (90% CI), while the standardized root mean square residual (SRMR ≤ 0.08) specified acceptable model fit ( 24 , 25 ).

Standardized estimates of regression coefficients (95% CI), by gender, as well as model fit, were produced in Mplus software (version 7.4, Muthen & Muthen, Los Angeles, CA, USA). All descriptive analyses were performed using R version 2.15.

## RESULTS

Of the study population (4814 men and 5582 women), 7.9% were night workers, mean age 49 years. On average, fasting glycemia was 110 mg/dL, 2-hour plasma glucose was 134 mg/dL, and glycated hemoglobin was 5.41%, the highest mean values being observed among men. Men also reported higher frequency of moderate or high physical activity ( Table 1 ). Snacking most frequently involved consumption of soda and pizza, while hamburger and hot dog were the foods least frequently consumed: 25.7% of participants reported never drinking soft drinks, 23.7% never ate pizza, 33.3% never ate salami, 72.2% never ate hot dog, 70.4% never ate hamburger, and 49.2% never ate fried savories.

**Table 1:** Workers’ characteristics, by gender – ELSA-Brasil, 2008-2010

	All N = 10,396	Men N = 4,814	Women N = 5,582
	**Mean (standard deviation)**		
Age (years)	49.1 (7.3)	49.5 (7.5)	48.8 (7.1)
Fasting glucose (mg/dL)	110.1 (28.9)	114.9 (32.5)	105.8 (24.7)
2-hour plasma glucose (mg/dL)	134.0 (52.1)	140.4 (58.5)	128.5 (45.1)
Glycated hemoglobin (%)	5.41 (0.94)	5.5 (1.1)	5.4 (0.8)
Waist measurement (cm)	90.6 (12.7)	94.8 (11.7)	86.9 (12.4)
Time on night work (years)	18.3 (8.4)	18.9 (8.8)	17.8 (8.1)

		**n (%)**	

Physical activity			
None/low	8054 (78.7)	3564 (75.2)	4490 (81.7)
Moderate	1254 (12.3)	654 (13.8)	600 (10.9)
High	925 (9.0)	522 (11.0)	403 (7.3)
Education			
Incomplete elementary school	510 (4.9)	346 (7.2)	164 (2.9)
Incomplete high school	635 (6.1)	383 (8.0)	252 (4.5)
Complete high school	3739 (35.9)	1640 (34.1)	2099 (37.6)
College degree	5512 (53.1)	2445 (50.8)	3067 (54.9)
Work schedule			
Day work	9578 (92.1)	4429 (92.0)	5149 (92.2)
Night work	818 (7.9)	385 (8.0)	433 (7.8)

Night workers were younger than daytime workers (48.4 [SD = 6.9] *vs* 49.2 [SD = 7.3] years), had less education (73.5% *vs* 44.7% completed high school), more reported no physical activity (84.4% vs 78.2%), had greater waist circumference (91.9 [SD = 12.6] *vs* 90.5 [SD = 12.7] cm) and more used medication for diabetes (13.3% *vs* 10.5%). Fasting glycemia (113.7 [SD = 35.4] *vs* 109.7 [SD = 28.3] mg/dL), glycated hemoglobin (5.4 [SD = 0.9] *vs* 5.5 [SD = 1.1] mg/dL), and glucose after glucose overload (133.5 [SD = 55.5] *vs* 139.8 [SD = 58.6] mg/dL) were higher among night workers.

The model presented in this paper refers to the relationships between night work, health behaviors, abdominal fat and glycemic levels, which displayed acceptable model fit. The model estimated ( Figure 1 ) represents the direct and indirect effects of night work on GLIC. The factor loads for latent variable “GLIC” were positive, with significant, high values for men and women. The factor load indicates that the increase in each of the variables (fasting glycemia, glucose after glucose overload, and glycated hemoglobin) leads to an increase in the latent variable “GLIC”. The variable with the highest factor load for this construct was fasting blood glucose (0.908 for men and 0.902 for women). A similar pattern was observed for the latent variable “Snacking” ( Table 2 ), although factor loads were lower, as expected for food consumption data, as these variables are difficult to measure accurately.

**Table 2:** Standardized estimates of the association between night work and glycemic levels using structural equation model, by gender – ELSA-Brasil (2008-2010)

	Standardized coefficients (95% CI)	

	Men	Women
Measurement model		
Glycemic levels (GLIC)		
Fasting glucose (FG)	0.912 (0.894; 0.931)***	0.908 (0.885; 0.930)***
2-hour plasma glucose (GSG)	0.869 (0.850; 0.887)***	0.866 (0.838; 0.894)***
Glycated hemoglobin (HbA1c)	0.797 (0.773; 0.820)***	0.776 (0.743; 0.809)***
Snacking		
Soda	0.451 (0.424; 0.477) ***	0.485 (0.458; 0.513) ***
Pizza	0.430 (0.406; 0.454) ***	0.454 (0.429; 0.480) ***
Ham/mortadella/salami	0.438 (0.414; 0.461) ***	0.465 (0.439; 0.490) ***
Hot dog	0.458 (0.430; 0.486) ***	0.494 (0.467; 0.522) ***
Hamburger	0.439 (0.410; 0.467) ***	0.464 (0.436; 0.491) ***
Fried savories	0.394 (0.369; 0.419) ***	0.476 (0.447; 0.504) ***
Structural model		
Direct effects		
1. GLIC		
Night work (NW)	0.022 (-0.013; 0.057)	0.031 (0.004; 0.058)*
Waist circumference (WC)	0.180 (0.145; 0.214)***	0.210 (0.183; 0.238)***
Snacking	- 0.060 (-0.108; -0.013)**	0.008 (-0.020; 0.035)
2. SNACKING		
Physical activity	0.084 (0.046; 0.123)***	0.120 (0.091; 0.150)***
3. SODA		
Night work	- 0.020 (-0.048; 0.007)	- 0.049(-0.074; -0.024)***
4. WC		
Night work	0.030 (0.002; 0.058)*	0.026 (0.002; 0.051)**
Physical activity	- 0.100 (-0.126; -0.074)***	-0.083 (-0.109; -0.058)***
Snacking	- 0.148 (-0.195; -0.100)***	- 0.090 (-0.137; -0.043)***
Soda	- 0.070 (-0.105; -0.036)***	-0.153 (-0.186; -0.120) ***
5. PHYSICAL ACTIVITY		
Night work	-0.038 (-0.064; -0.012)**	-0.043 (-0.065; -0.021)***
Indirect effects		
NW→WC→GLIC	0.005 (0.001; 0.010)*	0.006 (0.001; 0.011)*
NW→SODA→WC→GLIC	0.000 (-0.000; 0.001)	0.002 (0.001; 0.002)**
NW→PHYS.ACTI→WC→GLIC	0.001 (0.000; 0.001)**	0.001 (0.000; 0.001)**
NW→PHYS.ACTI→DIET→GLIC	0.000 (0.000; 0.000)	0.000 (0.000; 0.000)
NW→PHYS.ACTI→DIET→WC→GLIC	0.001 (0.000; 0.001)*	0.000 (0.000; 0.001)**
NW→PHYS.ACTI→DIET→SODA→WC→GLIC	0.000 (0.000; 0.000)*	0.000 (0.000; 0.000)**
Total indirect effects	0.007 (0.001; 0.012) **	0.008 (0.003; 0.013)**
Total effects	0.028 (-0.007; 0.064)	0.039 (0.011; 0.067) **
Model goodness-of-fit		
RMSEA (90% CI)	0.048 (0.047; 0.050)	
CFI	0.920	
TLI	0.901	
SRMR	0.058	

CFI: comparative fit index; TLI: Tucker-Lewis index; RMSEA: residual mean square error of approximation; SRMR: standardized root mean square residual. p-values: *p < 0.05; **p < 0.01; ***p < 0.001.

The structural model estimated standardized coefficients, which represent the relation between explanatory and response variables, expressed in standard deviation (SD) units. Night work was observed to have significant direct effect on GLIC only in women – exposure to night work was associated with a 0.031 SD increase in GLIC factor levels. In both genders, significant indirect effects mediated by physical activity, waist circumference and/or snacking were observed.

The standardized effects of each variable analyzed, as well as the values related to model fit, are shown in Table 2 .

## DISCUSSION

This study showed that night work has a significant direct effect on glycemic levels only in women, and a significant effect mediated especially by waist circumference for both genders. Identification of the association between night work and higher glycemic levels can be seen as an important step in the discussion of mechanisms that possibly explain why exposure to night work may be a risk factor for developing diabetes.

Significant associations between night work, diabetes and risk factors for the disease have been observed in prior studies ( 1 , 11 , 26 ), including findings from ELSA-Brasil, in which significant results were observed only among women ( 9 ). In a cohort study where analyses were also stratified by sex, the association between night work and diabetes was once again restricted to women ( 3 ). However, a meta-analysis suggested a stronger association between shift work and DM2 in men than in women ( 11 ). Qualitative review argued that differences in prevalence of diabetes between genders may be attributed to psychosocial and biological factors, but studies of gender-specific influences of shift work on metabolism are scarce and still inconclusive ( 27 ). Also, the lack of studies exploring the interaction between gender and shift work makes it difficult to compare results ( 9 ). The statistically significant associations of night work with increased waist circumference and physical inactivity observed in this study are consistent with findings that point to the adverse influence of night work on lifestyle and health behaviors ( 28 , 29 ). It has been suggested that physical inactivity may result from the fatigue associated with unsocial working hours and from the difficulty of participating in sports activity groups ( 30 ). In relation to waist circumference, it has been suggested that aspects of food consumption and circadian maladjustment may help to explain the effect of night work on weight gain and increased abdominal obesity, and may lead to overweight and obesity ( 30 ).

Night workers tend to opt for quick, easy-to-prepare foods, fat- and carbohydrate-rich meals, frequently high in calories and low in nutritional content ( 31 , 32 ). In addition, altered meal times are expected to affect internal body rhythms, i.e., working at night would entail a conflict between meal times and the circadian rhythms of hunger and satiety, increasing the predisposition to weight gain ( 32 ). The physiological explanation of this phenomenon presupposes that nocturnal wakefulness as a result of work leads to lower leptin levels and higher ghrelin levels, as well as affecting other hormones and neuropeptides involved in the regulation of appetite ( 33 ). This relationship may be partly explained by the effect of sleep restriction on activation of the sympathetic nervous system, which inhibits secretion of leptin by adipocytes ( 34 ). In this study, however, no associations were observed between night work and unhealthy food consumption, as measured by frequency of snacking pizza, hamburger, hot dog, ham/salami/mortadella, fried savories, and soda. These results thus indicate that, in this population, the relationship between night work and metabolic outcomes mediated by snacking may be explained better by circadian rhythm misalignment than by the type of food consumed by night workers. A recent systematic review found no differences in caloric intake between day and night workers, suggesting that factors such as circadian disruption, mealtimes, and variations in energy metabolism at night may explain the higher prevalence of obesity in shift workers than in day workers ( 35 ).

The main advantage of this study was that it enabled the indirect effects of night work on glycemic levels to be estimated, with emphasis on waist circumference as having an important mediating role in this association. Although the indirect effects of night work on glycemic levels are also mediated by snacking and physical activity, the effects derive predominantly from waist circumference, since no direct effects of physical inactivity on glycemic levels were observed. Thus, the ability to estimate the specific effects of each variable also contributes to an improved understanding of these relationships.

Note that, although the modeling used here presupposed causal relations, the cross-sectional study design does not allow a causal sequence to be established. In other words, although the model presented good quality of fit indices by including the effect of physical activity on waist circumference and snacking, it is possible that, for example, anthropometric indicators and snacking have effects on the practice of physical activity. Also, the model estimated does not represent a complete causal model for the investigation of all possible ways that night work could influence diabetes. In this context, it is emphasized that initially the factors psychosocial job stress (demand-control model) and work hours were tested in the model, but in this study they did not vary with work schedule and glycemic levels.

Finally, the individuals classified as night workers were heterogeneous because of (i) possible classification errors as regards this exposure in the ELSA-Brazil population, (ii) the number of working nights, and (iii) the total hours of night shift work. Also, “shift worker”, by definition, comprises groups with different work patterns ( 36 ), i.e., workers on alternating shifts or fixed night shifts, which can result in different health risks. An additional strength of the present study is that it was possible to identify those among the day workers who had previously worked at night.

Given the lack of homogeneity in the exposure variable, it was expected, a priori, that the effects of night work on glycemic levels would not be very large. It is relevant to mention that we showed standardized coefficients in order to compare the magnitude of each variable analyzed. Most studies on this topic, using adjusted regression models on a traditional approach, have suggested associations independently of potential mediators. In the literature, no studies were identified investigating these relationships using structural equation modeling to explore whether they may be mediated by physical activity, snacking and waist circumference. Therefore, it is possible that the low estimates observed in this study derive also from the type of analysis, using a more complex model that allows dependent and independent variables to be investigated simultaneously. In line with this discussion, the small effect size, despite statistical significance, should be taken into consideration when attempting to extrapolate the results. However, clinical researchers should also consider that our results in combination with previous knowledge highlight the role of shift work on waist circumference, an intermediate risk factor for diabetes.

Given the complexity of relations among these variables, these should be explored later with models that address the various possible interrelationships. Different biological pathways, including hormonal changes and sleep-related factors, deserve to be evaluated in the future in order to investigate different effects related to health behaviors and circadian mismatch. In all, the analyses presented here, which are based on observational studies, can be seen as an important step towards understanding the pathways that link night work to diabetes.

Funding: the ELSA-Brasil baseline study was supported by Brazil’s Ministry of Health (Department of Science and Technology) and Ministry of Science and Technology (Study and Project Funding agency – FINEP and National Research Council-CNPq) (grants 01 06 0010.00 RS, 01 06 0212.00 BA, 01 06 0300.00 ES, 01 06 0278.00 MG, 01 06 0115.00 SP, and 01 06 0071.00 RJ). ASC was a CNPq post-doctoral research fellow (150551/2015-0).
